# Investigating life‐history traits of Steller sea lions with multistate hidden Markov mark–recapture models: Age at weaning and body size effects

**DOI:** 10.1002/ece3.6878

**Published:** 2020-12-30

**Authors:** Kelly K. Hastings, Devin S. Johnson, Grey W. Pendleton, Brian S. Fadely, Thomas S. Gelatt

**Affiliations:** ^1^ Division of Wildlife Conservation Alaska Department of Fish and Game Juneau Alaska USA; ^2^ NOAA Fisheries Alaska Fisheries Science Center Seattle Washington USA

**Keywords:** Alaska, body size, *Eumetopias jubatus*, life history, mammals, pinnipeds, population dynamics, reproduction, trade‐offs

## Abstract

The duration of offspring care is critical to female fitness and population resilience by allowing flexibility in life‐history strategies in a variable environment. Yet, for many mammals capable of extended periods of maternal care, estimates of the duration of offspring dependency are not available and the relative importance of flexibility of this trait on fitness and population viability has rarely been examined. We used data from 4,447 Steller sea lions *Eumetopias jubatus* from the Gulf of Alaska and multistate hidden Markov mark–recapture models to estimate age‐specific weaning probabilities. Maternal care beyond age 1 was common: Weaning was later for animals from Southeast Alaska (SEAK) and Prince William Sound (PWS, weaning probabilities: 0.536–0.648/0.784–0.873 by age 1/2) compared with animals born to the west (0.714–0.855/0.798–0.938). SEAK/PWS animals were also smaller than those born farther west, suggesting a possible link. Females weaned slightly earlier (+0.080 at age 1 and 2) compared with males in SEAK only. Poor survival for weaned versus unweaned yearlings occurred in southern SEAK (female survival probabilities: 0.609 vs. 0.792) and the central Gulf (0.667 vs. 0.901), suggesting poor conditions for juveniles in these areas. First‐year survival increased with neonatal body mass (NBM) linearly in the Gulf and nonlinearly in SEAK. The probability of weaning at age 1 increased linearly with NBM for SEAK animals only. Rookeries where juveniles weaned at earlier ages had lower adult female survival, but age at weaning was unrelated to population trends. Our results suggest the time to weaning may be optimized for different habitats based on long‐term average conditions (e.g., prey dynamics), that may also shape body size, with limited short‐term plasticity. An apparent trade‐off of adult survival in favor of juvenile survival and large offspring size in the endangered Gulf of Alaska population requires further study.

## Graphical abstract

Extended maternal care past age 1 was common in Steller sea lions, but ranged geographically (e.g., 0.54–0.86 were weaned at age 1); where offspring were smallest, weaning was later. Earlier weaning and larger offspring were associated with reduced adult female survival but were unrelated to population trends, suggesting this trait may be optimized for different habitats based on long‐term average conditions, that may also shape body size, with limited short‐term plasticity.

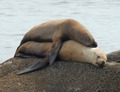

## 1 INTRODUCTION

Many life‐history traits (e.g., age‐specific reproductive effort and mortality; body size and growth; number, size, and sex of offspring; lifespan) interact to determine which genetic and phenotypic traits are passed on to future generations and the viability of animal populations. Within phylogenetic and biological constraints, life‐history traits occur as a suite of biological processes and behaviors that are collectively under selection to optimize the number of offspring produced per individual for a given set of environmental and biological conditions (Stearns, 1992). The number of offspring produced that also contribute to future generations determines an individuals' fitness. Understanding how life‐history strategies respond to changing environmental conditions is important for accurate modeling of wildlife populations.

Trade‐offs commonly occur among life‐history traits such that allocation to one trait may be detrimental to another; the trade‐off between current reproduction and future survival and reproduction particularly shapes female reproductive strategies (Stearns, 1989). In female mammals, reproductive strategies include variations in age‐ or size‐specific frequencies of reproduction, litter size, body size of offspring, and levels and length of time of maternal care (Clutton‐Brock, 1991; Stearns, 1992), including direct provisioning of food, physical protection, and teaching vital skills. Food intake is the most fundamental factor influencing mammalian reproduction (Bronson, 1985) and may influence all aspects of these reproductive strategies. In mammals, maternal care strategies may be particularly important to female fitness because the cost of lactation is much higher than the cost of gestation (Gittleman & Thompson, 1988). This is especially true of female pinnipeds (seals, sea lions, fur seals, and walruses) in which litter size is constrained to one (twins are produced only very rarely: Gelatt et al., 2001; Maniscalco & Parker, 2009; Spotte, 1982) and the energy cost of lactation is particularly high (Oftedal et al., 1987).

Among pinnipeds, eared seals (family Otariidae, including 9 fur seal species and 6 extant sea lion species) have the highest energy costs of both existence and lactation (reviewed by McHuron et al., 2017a). They are "income" breeders (Jönsson, 1997) that must feed while lactating during a long and variable lactation period (4 months–3 + years), and so have the ability for simultaneous gestation and lactation with slow offspring growth rates (Costa, 1991). Because of the reduced energetic efficiency of this reproductive strategy (due to a lower ratio of energy allocated to offspring growth vs. maintenance compared with capital breeders [described below] and to the need for continual feeding over a long period with potentially variable food conditions [reviewed by Stephens et al., 2014]), otariid individuals and populations are particularly vulnerable to disturbances in their energy balance (McHuron et al., 2017b; Soto et al., 2004). The contrasting reproductive strategy, "capital" breeding, occurs in earless seals (family Phocidae) that fast during a short lactation period (4–50 days, Oftedal et al., 1987), converting stored blubber acquired during gestation (supplemental feeding during late lactation occurs in some species; Bowen et al., 2001; Lydersen & Kovacs, 1999; Wheatley et al., 2008), producing high neonatal pup growth rates. Capital and income breeding occur throughout animal and plant species (Jönsson, 1997), but pinniped species are unique in the range of their use of storage for offspring provisioning (Stephens et al., 2014).

Energetic costs are greatest during late lactation (McHuron et al., 2017a, Winship et al., 2002; but see Melin et al., 2000) leading to high rates of late‐term abortions and lower birth rates in some otariid species (Gibbens et al., 2010; Higgins & Gass, 1993; McKenzie et al., 2005; Pitcher et al., 1998), particularly during periods of poor food availability (Pitcher et al., 1998). Some otariid species can extend lactation beyond 1 year at the expense of new offspring production, suggesting a trade‐off between offspring quality and quantity. This “bet‐hedging” strategy is thought to maximize fitness in an unpredictable environment (Balme et al., 2017; Stearns, 1976). In otariids capable of lactation periods > 1 year, the production of new offspring may be reduced through late‐term abortion but also by the tendency to favor the dependent juvenile after a new pup is born (Maniscalco & Parker, 2009). During the breeding season, few (1.9%–3.7%; Maniscalco & Parker, 2009, Alaska Department of Fish and Game ADFG, *unpublished data*) Steller sea lion *Eumetopias jubatus* females with pups are also observed with a dependent juvenile; this rate may be higher in other species (7–>20%, Majluf, 1987; Trillmich & Majluf, 1981; Trillmich & Wolf, 2008).

Although extended lactation periods have been observed in some otariids, precise estimates of weaning age are rare, largely because of the difficulty in obtaining such estimates. For observational studies, individually marked animals often must be followed at large temporal and spatial scales. Dependent juveniles and pups only intermittently suckle, such that these studies require multiple sightings per individual and probabilistic models, only recently developed (Harris, 2016; Johnson et al., 2016; Laake et al., 2014), to distinguish the observational noise processes of animal detection and correct state assignment (weaned vs. unweaned) from the biological processes of survival and weaning.

Steller sea lions are the largest otariid species ( 2‐3 times larger than any of the other 5 extant sea lion species) and the most polarly distributed sea lion species, with population abundance historically centered in the northern Gulf of Alaska through the Aleutian Islands (Merrick et al., 1987, Figure 1). Breeding aggregations occur around the North Pacific Rim from California through Russia and Japan, and some males range seasonally northward as far as the Bering Strait (Merrick et al., 1987, ADFG *unpublished data*). Energetic costs of lactation are high in this species due to large body size, relatively small pup birth mass relative to maternal mass, fast pup growth rates, and low proportions of milk fat compared with other otariids (Boness & Bowen, 1996; Brandon et al., 2005; Schulz & Bowen, 2004). For example, energy demand is 70% greater for lactating than nonlactating females (Winship et al., 2002).

**Figure 1:**
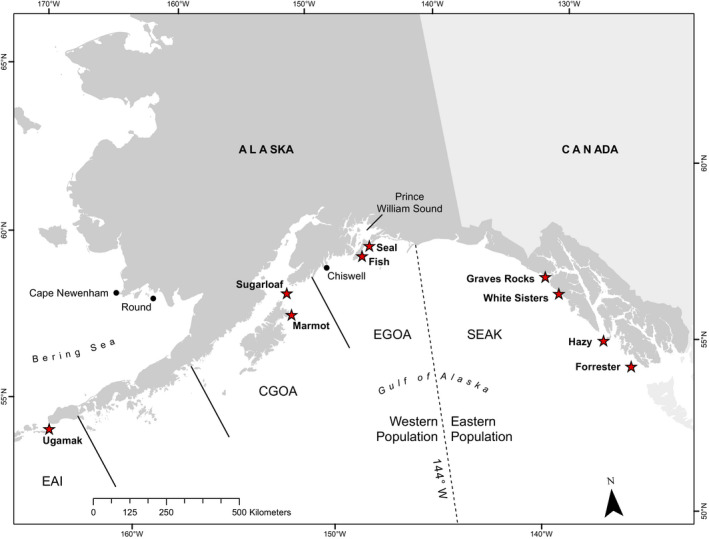
Map of the study area where Steller sea lions (*Eumetopias jubatus*) were marked and resighted from 2000 to 2018. Pups were marked at nine rookeries in red stars: Ugamak Island, Marmot Island, Sugarloaf Island, Fish Island (Prince William Sound), Seal Rocks (Prince William Sound), Graves Rocks, White Sisters, Hazy Islands, and Forrester Islands. 144° W marks the boundary between the western and eastern populations. EGOA, CGOA, and EAI are large management areas: eastern and central Gulf of Alaska and the eastern Aleutian Islands (Fritz et al., 2016). Data from animals born in these three areas are referred to as "Gulf of Alaska" animals versus "Southeast Alaska" in our study

The lactation period is long and variable in Steller sea lions; weaning may occur from 1 to 3 + years of age (Pitcher & Calkins, 1981), with significant numbers weaning at >1 year (perhaps > 40%–50%: Trites et al., 2006, York et al., 2008). A seasonal peak in weaning may occur in April–May, as females return from haul‐outs and foraging areas to rookeries to give birth and breed (Loughlin et al., 2003; Raum‐Suryan et al., 2004; Trites et al., 2006). Age‐specific weaning probabilities are not yet precisely estimated for this species over a significant spatial scale. Estimates of weaning probabilities from mark–recapture models are available for a sample of animals born at a small rookery in the eastern Gulf (~100 pups produced per year, Maniscalco, 2014; Chiswell Island, see Figure 1). However, estimates are needed over a larger spatial scale, as the dynamics of Steller sea lion populations respond to localized conditions over relatively small‐spatial scales (Lander et al., 2009; O’Corry‐Crowe et al., 2006; Sinclair & Zeppelin, 2002; York et al., 1996) and are likely dependent on animal density in relation to food supply (Hastings et al., 2011; Jemison et al., 2018).

Understanding life history and maternal care strategies is valuable to conservation and management of this species. Severe population declines (>70%) over a large portion of their range resulted in listing under the U.S. Endangered Species Act (Merrick et al., 1987, U.S. Federal Register, 1997). Despite the declines' severity and acuteness (occurring over ~25 years from the mid–late 1970s until ~2000–2003; Fritz et al., 2016), conclusive evidence that food limitation was the primary driver is lacking. The decline coincided with a dramatic, and at least partially climate‐induced shift in nearshore fish assemblage in the North Pacific Ocean in the late 1970s and early 1980s (Anderson & Piatt, 1999, Hare & Mantua, 2000, but see Fritz & Hinckley, 2005) and large‐scale development of commercial fisheries in the Gulf of Alaska (Alverson, 1991; Hennen, 2006). Some potential evidence for food limitation was observed (smaller body size of nonpups, reduced body condition of lactating females also producing lower birth rates, slower body growth coupled with later weaning ages, and longer at‐sea foraging trips for juveniles; Calkins et al., 1998; Call et al., 2007; Pitcher et al., 1998; York et al., 2008), but some expected responses to poor food conditions were not (reduced pup and juvenile body sizes, and longer female foraging trips; Brandon et al., 2005; Merrick et al., 1995; Milette & Trites, 2003; Rea et al., 2016). Also, demersal fish abundance was high when the population continued to decline (Mueter & Norcross, 2002) and energy content and composition of diet could not be linked to health and condition of juveniles (Calkins et al., 2013).

How female Steller sea lions cope with food shortages remains largely unknown, and studies of the behavior and health of animals during vulnerable periods (winter and spring; Winship et al., 2002) or at the most vulnerable ages (Benton et al., 1995), such as just after weaning, may be critical to fill this knowledge gap. A large dataset is now available to study weaning patterns: >9,000 individuals were permanently marked as neonates on their natal rookeries over the past several decades in Russia and the United States by various agencies. Data from these marked, known‐aged animals have provided precise and spatially diverse estimates of age‐specific survival and movement probabilities (Altukhov et al., 2015; Fritz et al., 2014; Hastings et al., 2011; Jemison et al., 2018; Maniscalco, 2014; Pendleton et al., 2006; Wright et al., 2017). However, other life‐history traits have not yet been fully evaluated. Here, we used data from animals born from 2000 to 2016 and mark–recapture models to estimate age‐specific weaning probabilities for animals born at nine rookeries in Southeast Alaska and the Gulf of Alaska (three large management areas to the north and west of Southeast Alaska, see Figure 1), with a particular interest in spatial and sex‐specific patterns. We also examined the relationships between spatial variation in age‐specific weaning probabilities and spatial variation in population trends and other life‐history traits (juvenile and adult female survival and neonatal body size), to ascertain whether Steller sea lion females may adapt maternal care and life‐history strategies to environmental conditions (e.g., if earlier or later weaning occurred in areas where population growth, neonatal body size, and juvenile survival were highest, and whether these patterns were associated with improved or reduced adult female survival, suggesting important trade‐offs among these traits).

## 2 MATERIALS AND METHODS

### 2.1 Animal marking and resighting

**Figure 2:**
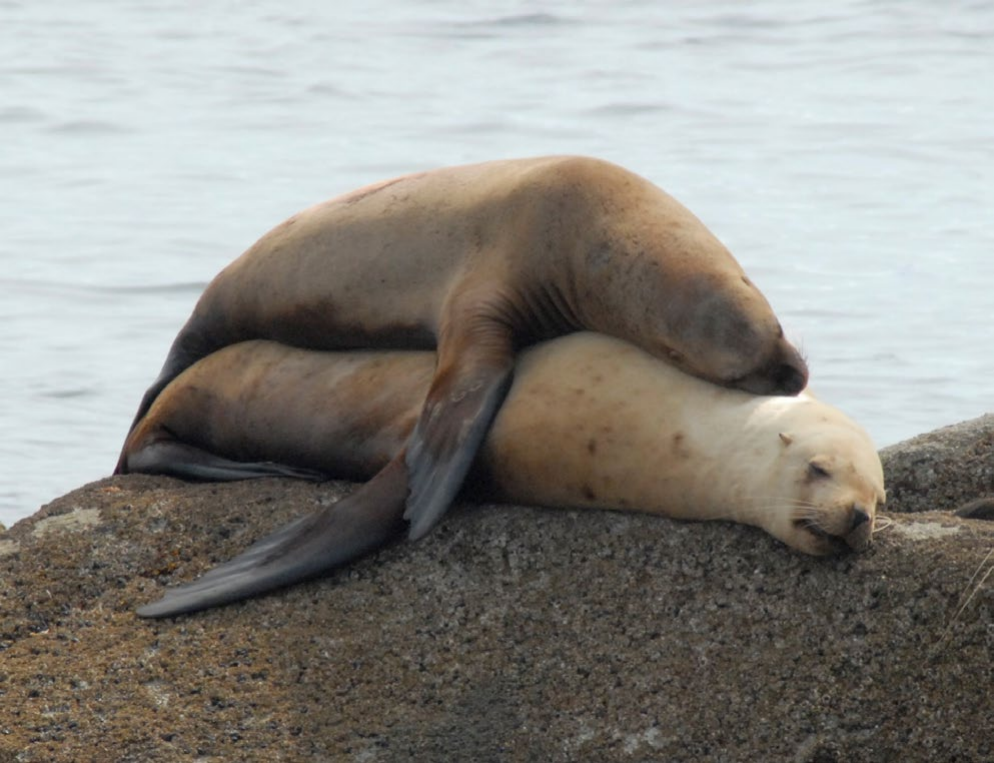
Example of a Steller sea lion juvenile laying squarely on top of its mother, a behavior that is distinctive to mother–offspring pairs and considered definitive of an unweaned juvenile. Photo credit: L. Jemison/Alaska Department of Fish and Game

Steller sea lion pups were captured, anesthetized, and hot‐branded with unique alphanumeric combinations at 2–4 weeks of age at nine rookeries in the Gulf of Alaska and Southeast Alaska from 2000 to 2016 (*n* = 4,447; Table 1, Figure 1). At the time of marking, pups were weighed to the nearest 0.1 kg and a skin sample was collected from the webbing of a hindflipper to provide a DNA sample. Resightings and photographs of branded animals were collected annually from May to August, during standardized surveys and from miscellaneous sightings that covered the geographic range of the species from California through Russia and into the Bering Sea (Fritz et al., 2014; Hastings et al., 2011; Jemison et al., 2018; Wright et al., 2017). During brand resighting surveys, juveniles were considered "unweaned" if they were observed suckling. We also noted if juveniles were laying squarely on top of their mother (Figure 2), a behavior that is observed rarely but is distinctive to mother–offspring pairs and considered definitive of an unweaned juvenile. Other behaviors that possibly indicated a juvenile–mother pair were recorded (behavioral interaction between female and juvenile) but were not reliable indicators of a dependent juvenile with mother, as extensive social interaction is common in sea lions. Branded animals could be observed multiple times per summer, demonstrating a variety of behaviors during different observations (of branded juveniles in this study: percentages seen 1, 2, 3, 4, and 5 + times per summer were 51, 20, 8, 4, and 17, respectively). In the analyses, we used only data of animals whose identities were photographically confirmed by comparison to our photograph library of all animals (included 84% of resights).

**Table 1:** Number of Steller sea lion pups branded by the Alaska Department of Fish and Game and the National Marine Fisheries Service from 2000 to 2016 in the Gulf of Alaska, western population, and Southeast Alaska, eastern population

Natal rookery	Birth year												
	2000	2001	2002	2003	2004	2005	2008	2009	2010	2011	2014	2016	Total
Southeast Alaska													
Forrester Islands		286	141	291	277								995
Hazy Islands		213		101		225							539
White Sisters			127		94	147							368
Graves Rock			50			43						70	163
Gulf of Alaska													
Fish Island, PWS		32											32
Seal Rocks, PWS		75		100		80							255
Sugarloaf Island	151		105		110		93		100		91		650
Marmot Island	107		89		75		85		78		100		534
Ugamak Island		175		150		200		188		198			911

Note

PWS = Prince William Sound. See Figure 1 for rookery locations.

Because resighting schedules differed for the Gulf of Alaska and Southeast Alaska, datasets were created and modeled separately. In Southeast Alaska, all rookeries and haul‐outs were observed in a standard manner each year during 1–3 large‐scale dedicated boat‐based trips from 2002 to 2018. Geographic coverage was consistent among years, and the dates of annual boat surveys varied from mid‐ to late June during 2002–2004, mid‐July during 2005–2007, and late June to early July during 2008–2018. A field camp at the Forrester Island Rookery Complex was also staffed from late May to at least early July every year, and observations were collected daily at Lowrie Island and weekly after 2005 at other islands at Forrester Island Complex. From 2001 to 2004, 2–3 surveys were conducted per summer on consecutive days at rookeries and most often 1 survey per summer at haul‐outs. Since 2005, effort at rookeries increased to 4–6 surveys per summer on consecutive days at rookeries (these multiple surveys per rookery each summer provided the majority of repeated sightings per individual for both datasets).

In the Gulf of Alaska, animals were resighted annually in spring or summer from 2001 to 2016. Resighting effort varied spatially and temporally over the years. In most years, most rookeries and haul‐outs from Prince William Sound to Ugamak Island were surveyed at least 1–2 times either in late spring–early summer or in late summer. The haul‐outs and rookeries around Ugamak Island were infrequently surveyed, especially in later years. A few areas of daily resighting occurred: at Chiswell Islands and nearby haul‐outs in the eastern Gulf (by the Alaska SeaLife Center, Maniscalco, 2014), and at Round Island (May–August) and Cape Newenham in the Bering Sea (Figure 1). Like the Forrester Island Complex, annual summer field camps from May–August at Ugamak and Marmot Islands provided consistent, high resighting effort in all years at these sites through daily surveys. Resighting effort at the three other rookeries where pups were branded (Seal Rocks, Fish Island, and Sugarloaf Island) included 1–2 surveys per site per spring or summer from 2001 to 2006, with increased effort (4–10 + surveys per site per summer, on consecutive days, in July) from 2007 to 2016. Therefore, besides the two areas of high resighting effort mentioned (Figure 1), the rookeries that were heavily monitored were also the natal sites of marked pups. Procedures for handling and observing sea lions were approved by Institutional Animal Care and Use Committees and by permits issued by the U.S. National Marine Fisheries Service (NMFS) to the ADFG and the NMFS Marine Mammal Laboratory (MML).

### 2.2 Statistical modeling: Capture histories

To estimate weaning rates, we created capture histories from resighting data and fit multivariate state‐based Cormack–Jolly–Seber (CJS) models that allowed imperfect state detection. These models are formulated as a hidden Markov model so that maximum‐likelihood estimation can be used for parameter inference (Johnson et al., 2016; Laake et al., 2014). Codes used in capture histories were "0" if an animal was not seen on an occasion, "S" if seen suckling (or laying on top of a female) on that occasion, and "u" (unknown) if seen but not seen suckling that occasion. All pups were assigned an "S" code in their birth year. In this study, we defined the weaned state as the time when suckling completely ceased.

It was possible to create capture histories using the most definitive behavior observed to summarize multiple sightings of juveniles for an occasion (i.e., assign an "S" if ever seen suckling). However, initial testing demonstrated that biased parameter estimates resulted from not accounting for individual heterogeneity in the number of times animals were seen. This was particularly due to the probability of positively detecting the unweaned state increasing with the number of times an animal was seen (i.e., the more times a juvenile was seen, the more likely it was that an "S" would be recorded in its' capture history). We accounted for this effect by creating capture histories using a robust design (Williams et al., 2001) with 1–2 primary occasions per summer and 5 secondary occasions within each primary occasion. The robust design was used to allow the probability of positively identifying the unweaned state to increase with the number of observations of the animal within a season (see the following section for more explanation based on the statistical model). If a juvenile (ages 1–3 years) had *x* ≤ 5 observations, the secondary capture history included those observations followed by 5−*x* 0s. However, if more than 5 observations were available for the secondary capture history, 5 were randomly selected except that observations at haul‐outs rather than rookeries were favored to counterbalance the high resight effort at natal sites. For example, a capture history for an animal observed 3 times at age 1, 7 times at age 2, and not observed at age 3 could be (for these 4 primary occasions) S0000 (birth year), uuS00 (at age 1), uuuSu (at age 2 where 5 of 7 observations were chosen randomly) and 00000 (at age 3). The secondary occasions were included only for years in which marked juveniles were in the population. All animals observed in a primary occasion had data for the first secondary occasion; adults (ages 4 + years) could have data *only* in this first secondary occasion. A time‐varying individual covariate ("ns," number of times seen) was included with each animals' capture history to indicate the number of times the animal was observed each primary occasion as a juvenile; ns was defined to be 1 for all adults when observed.

In Southeast Alaska, the total number of occasions in capture histories was 54:18 primary annual occasions (2001–2018) and 5 secondary occasions only for 9 years (2002–2008 and 2017–2018, when marked juveniles were in the population; 9 * 5 + 9 occasions). For Southeast Alaska animals, we created capture histories using only data collected from June 20 to August each year. Although weaning rates may peak in April–May (Loughlin et al., 2003; Raum‐Suryan et al., 2004; Trites et al., 2006), juveniles may continue to wean throughout the summer depending on the pupping status and behavior of the mother (Maniscalco & Parker, 2009). Therefore, we standardized the seasonal cutoff point for estimating annual weaning rates to >20 June to include most of the data available for Southeast Alaska animals, and because we expected this date to be after the time of peak weaning and after the majority of new pups have been produced (Kuhn et al., 2017; Pitcher et al., 2001).

Due to temporal and spatial variation in resight coverage in the Gulf of Alaska (e.g., some areas in some years may have only sightings in May or early summer before new pups are produced), capture histories of Gulf of Alaska pups included two primary occasions per summer to allow two cutoff points in time: 1 May–19 June (EARLY) and 20 June–August (LATE, the seasonal cutoff used for Southeast Alaska animals). For this dataset, the total number of occasions in capture histories was 161:16 annual occasions (2001–2016) with EARLY and LATE seasons, each with five secondary occasions (16 * 2 * 5 = 160) plus one initial occasion for the release of the 2000 cohort.

### 2.3 Statistical modeling: Parameter estimation

We used the R package marked (model "mvmscjs," Laake et al., 2013; R Core Team, 2019) to estimate parameters and to calculate AIC Weights for model selection (Burnham & Anderson, 2002). Parameters in models included nuisance parameters: animal resighting rate (*p*) and state detection rate (i.e., weaned or unweaned, *δ*), and parameters of interest: survival probability (*Φ*) and the probability of changing weaning state (*ψ*), specifically the probability of weaning (*ψ*_S:W_). For all secondary occasions, *Φ* was fixed to 1.0 (i.e., no mortality or emigration between secondary periods). For animals seen only one time (ns, number of times seen = 1) in a given primary occasion, *p* was fixed to 0 (i.e., resighting impossible) for the last 4 secondary occasions. For juveniles with ns > 1 (seen *x* > 1 time per year), *p* was fixed to 1 (i.e., resight certain) for secondary occasions > 1 and ≤ *x*, and to 0 for secondary occasions > *x*. By constraining the detection process in this way, *p* is interpreted as the probability the animal was seen at all in the primary occasion, as with a traditional CJS model. The state detection parameter *δ* was the probability of detecting the "unweaned" state (state S) on any given secondary observation. For *δ*, we fixed the probability of detecting the weaned state (state W) to 0 because definitively observing this state was impossible (only "S" and "u" may be observed). By modeling the probability of an accurate "S" detection (as opposed to observing "u") for each secondary occasion, the probability of accurately classifying a juvenile's state within the primary period was 1 − (1 − *δ*)^ns^. Thus, as ns went from 1 to 5, the probability approached 1 geometrically. This result was the reason for using the robust design within this multistate model.

For *ψ*, only the probability of transitioning from states unweaned to weaned (*ψ*_S:W_) was estimated. Probability of transitioning from state W to S was fixed to 0, and from state W to W to 1, as weaned was an absorbing state. For the Gulf of Alaska analyses, the two primary occasions per summer were considered open with respect to weaning (*ψ*_S:W_) but closed with respect to *Φ* (*Φ* was fixed to 1.0), because we expected that *Φ* was ~1.0 for this short time period. For analysis of each dataset, we used a single *δ* model with constant *δ* for all individuals and occasions.

For the Southeast Alaska analysis, our base *p* model was age * sex + year + nr_adults, where age was 13 (1–12 and 13 + years) and where nr = natal rookery and nr_adults was the effect of nr at age 4 + for females and age 6 + for males (Hastings et al., 2018). The base model also included an effect of maternal genetic lineage (as determined from mitochondrial DNA, *mtHap*) on probability of seeing an animal at age 1, for animals born at Graves Rocks and White Sisters (Figure 1, Hastings et al., 2020). Two populations of Steller sea lions are recognized in Alaska due to differing demographics and distinct genetic differences, an eastern and a western population (O'Corry‐Crowe et al., 2014, Figure 1). Graves Rocks and White Sisters are productive, new rookeries in northern Southeast Alaska (Mathews et al., 2011; Pitcher et al., 2007) within a mixing zone of animals from the genetically distinct populations (O'Corry‐Crowe et al., 2014, Figure 1), and survival and behavior vary with maternal lineage for these pups (Hastings et al., 2020). *mtHap* was available for 420 of the 531 pups (Hastings et al., 2020), so a portion of these pups were of unknown maternal lineage. We included *mtHap* (*mtW* = western, *mtE* = eastern, and *mtU* = unknown) to determine whether weaning probabilities differed based on this variable, while accounting for animals with uncertain lineage. In addition to the base model, we fit one additional *p* model that included an effect of weaning state on *p* for juveniles; we expected that weaned juveniles may have lower *p* than unweaned juveniles (2 *p* models fit).

For the Southeast Alaska analysis, we modeled age effects on *ψ*_S:W_ as 4 (ages 0, 1, 2, and 3+) or 3 categories (ages 0 = 1, 2, and 3+). We included *ψ* models with effect of natal region (regS = south, born at rookeries Forrester or Hazy Islands, vs. regN = north, born at White Sisters or Graves Rocks; Figure 1) at ages 0–2, because we expected that factors responsible for lower survival of regS animals (Hastings et al., 2011) may also affect weaning probabilities. We also included *ψ* models with effects of sex and *mtHap* at ages 0–2 (13 *ψ* models fit). For *Φ*, our base model was sex * age + nr, where ages were 4 categories for females (0, 1, 2, and 3+) and 5 categories for males (0, 1, 2, 3–8, and 9+; Hastings et al., 2011, 2018). The base model included *mtHap* effect on first‐year survival for regN animals (Hastings et al., 2020). In addition to the base model, we included models with survival effects for animals weaning at ages 1–2 differing based on sex, *mtHap,* and natal region (11 *Φ* models fit).

Finally, we fit models that included the effects of neonatal body mass on first‐year survival, survival of weaned yearlings, and weaning probability to age 1. Because pups ranged in age (most between 2 and 4 weeks at the time of branding; Hastings et al., 2011, Maniscalco, 2014), body mass at branding was adjusted by capture date and mean birth date for each rookery to allow a rough adjustment for age at branding. Pups grow quickly as neonates (Brandon et al., 2005; Maniscalco, 2014), mean birth dates may differ up to 10 days among Alaskan rookeries (Pitcher et al., 2001), and dates of capture for our study animals differed up to 14 days (from 23 June to 7 July, and from 14 to 29 days after the rookery‐specific mean birth dates). To adjust mass for age effects, we fit generalized linear models by simplifying the most complex model, mass = nr * sex + population * day, where day was number of days between the capture date and the rookery‐specific mean birth date. Mean birth dates for each rookery (assumed constant over years) were 8, 9, 13, 14, and 4 June for Ugamak, Marmot, Sugarloaf, Prince William Sound, and Southeast Alaska rookeries, respectively (Kuhn et al., 2017; Pitcher et al., 2001). Where no estimate of mean birth date was available, we used the date of the closest rookery (Seal Rocks = Fish Island in Prince William Sound; Southeast Alaska rookeries assumed equal to the Forrester Island rookery). Day effect was allowed to differ between eastern and western populations because pup growth rates may differ between populations (with possibly slower growth in the east than in the west; Brandon et al., 2005). All models included sex effects as larger mean masses of male than female pups are firmly established (Brandon et al., 2005; Merrick et al., 1995; Rea et al., 2016). Coefficients from the best model, determined by AIC weight, were used to recalculate each individual's mass using mass – day * $\hat{\beta }$_day_. Adjusted mass (included as the deviations from the sex‐specific means in kg) was added to the best survival model as an individual covariate relating mass to survival as a linear trend or as a nonlinear pattern fit with a *b‐*spline smooth (*df* = 3–6, Hastie, 1992).

For the Gulf of Alaska analyses, our base *p* model included age class(ac) * sex * region * survey, with regions (a) Ugamak Island (management area eastern Aleutian Islands, Figure 1), (b) central Gulf (Marmot Island, Sugarloaf Island), and (c) Prince William Sound (Fish Island, Seal Rocks in the eastern Gulf, Figure 1), and ac = juveniles (1–3 years) and adults (4 + years). Models including a weaning effect on *p* of juveniles were also fit (8 *p* models fit). For *ψ,* up to 4 age‐based weaning probabilities were fit: a0 (age 0 to EARLY 1), a1 (LATE 1 to EARLY 2), a2p (after LATE 2), and a01summer (EARLY 1 to LATE 1 = EARLY 2 to LATE 2). We included *ψ* models that had common parameters for a0 and a1, and also models with region, natal rookery (nr), and sex effects on the main periods, a0 and a1 (7 *ψ* models fit). Our base *Φ* model followed Fritz et al. (2014): sex * age * nr where age had 4 categories for females (0, 1, 2, and 3+) and 5 categories for males (0, 1, 2, 3–8, and 9+; an additional senescent age class was included for males based on the results in Hastings et al., 2018). Additional *Φ* models included a survival effect of weaning at age 1 or 2, possibly varying with nr or region (10 *Φ* models fit). Finally, the effects of neonatal body mass on parameters were fit as in the Southeast Alaska analysis.

To produce comparable seasonal cutoff points for weaning (>20 June) for the two sets of analyses , we multiplied estimates for the early and late periods for the Gulf of Alaska data for the ages 0–1 and 1–2. For example, $\hat{\psi }$_S:W,0–1L_ was calculated as $\hat{\psi }$_S:W,0–1E_ + ((1‐$\hat{\psi }$_S:W,0–1E_)* $\hat{\psi }$_S:W,1E–1L_), where 0 and 1 = age 0 and age 1 and E and L = EARLY and LATE seasons, respectively. The cumulative proportion weaned for all pups born in both analyses was then calculated at age 1 as $\hat{W}$_1_ = $\hat{\psi }$_S:W,0–1L_. For ages *x* = 2–4, this value was calculated as $\hat{W}$_x_ = $\hat{W}$_x‐1_ + ((1 − *Ŵ_x‐1_*) * $\hat{\psi }$_S:W,__x‐1 to x_).

Finally, we used errors‐in‐variables linear regression, using a maximum‐likelihood procedure following Murphy & Van der Vaart (1996), to examine whether the proportion weaned at ages 1 and 2 and the survival effects of weaning were correlated with other life‐history parameters. This method was used to account for measurement error in both the predictor and response variables. Parameters we evaluated included juvenile female survival probability to breeding age (cumulative survival from 0 to 4 years), adult female survival (cumulative survival over 4 years, e.g., from 4 to 8 years), average female neonatal body mass (at 30 days past the mean birth date) adjusted for age/date effects, trend in pup counts at rookeries (from Jemison et al., 2018), and regional trends in numbers of pups and nonpups from summer aerial surveys from 2003 to 2015 for the Gulf of Alaska and from 1985 to 2015 for Southeast Alaska (Fritz et al., 2016; trends were similar in Southeast Alaska before and after 2003; see Figure 6g and 7g in Fritz et al., 2016). Confidence intervals [*CI*] for derived values (cumulative survival and weaning probabilities) were approximated using a multivariate normal parametric bootstrap with the mean equal to the maximum‐likelihood estimate and the covariance matrix equal to the negative Hessian of the log‐likelihood function, following Johnson et al. (2016).

## 3 RESULTS

### 3.1 Body mass adjustments based on capture date/age

**Figure 3:**
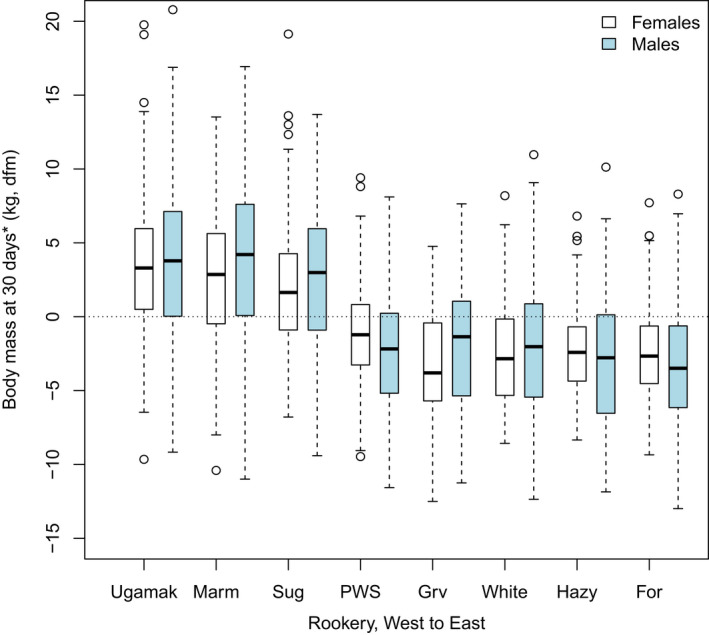
Variation among natal rookeries in the estimated body mass of Steller sea lion pups at 30 days *after the mean rookery‐specific birth date (in kg). Body mass is presented as deviations from the sex‐specific means for the whole dataset (dfm). Estimates of the population‐specific slopes for the variable "day" (number of days between the capture date and the rookery‐specific mean birth date) from model 1 in Table 2 were used to adjust mass measurements at the time of capture to 30 days after the rookery‐specific mean birth date. Data were summarized and plotted using box plot in R. Boxes include the median, the 1st and 3rd quantiles, the whiskers (extending to the most extreme data points which are < the range * the interquartile range), and outliers

Mass varied with sex * nr in our sample, in the expected manner (Brandon et al., 2005; Merrick et al., 1995; Table 2). Neonatal masses were 14%–20% larger at rookeries farther west than in Prince William Sound and Southeast Alaska at 30 days after the rookery‐specific mean birth date, due to higher Δ mass/day in the west ($\hat{\beta }$_day_ = 0.337, *95% CI [0.259, 0.415]*) than in the east ($\hat{\beta }$_day_ = 0.164 *[0.084, 0.244],* Figure 3).

**Table 2:** Model selection results for effects of capture date/age, natal rookery (nr), and sex on body mass of Steller sea lion pups in Southeast Alaska and the Gulf of Alaska

Model#	Model	Npar	AIC	ΔAIC	AIC Weight
1	nr * sex + population * day	19	464.1	0.0	0.97
2	nr * sex + day	18	471.2	7.1	0.03
4	nr + sex + population * day	12	488.8	24.6	0.00
5	nr + sex + day	11	496.9	32.8	0.00
3	nr * sex	17	546.5	82.4	0.00
6	nr + sex	10	568.4	104.3	0.00
7	sex + population * day	5	811.3	347.2	0.00
9	sex	3	1,465.3	1,001.2	0.00
8	sex + day	4	1,466.1	1,002.0	0.00

Note

Population was eastern versus western (see Figure 1). Day was number of days between the capture date and the mean rookery‐specific birth date.

### 3.2 Southeast Alaska

The average number of branded juveniles resighted per year in Southeast Alaska was 220, ranging from 92 to 329. When the most definitive behavior was summarized per summer per animal for the Southeast Alaska dataset, the numbers of animals best observed as "S" (i.e., definitively unweaned, such as suckling) at ages 1, 2, 3, and 4+ (*n* = 215 summer * animal) were 160 (74%), 51 (24%), 4 (2%), and 0, respectively. Observing juveniles laying on top of females (Figure 2) was rare and contributed 2.5% of the definitively unweaned sightings. The probability of observing an unweaned juvenile suckling in a single encounter ($\hat{\delta }$), definitively determining it was unweaned, was 0.226 *[0.192, 0.265]*. This implies that the probability of detecting the unweaned state was 0.226 for 1 observation within a primary occasion and 0.722 for ns = 5. Thus, there is a marked increase in detection when observing juveniles for ns > 1. Resighting probabilities $\left(\hat{p}\right)$ averaged 1.96 times higher for unweaned than weaned juveniles (Table 3b, model 14 vs. 10, ΔAIC > 18).

**Table 3:** Model selection results for Southeast Alaska analysis

Model#	Model	Npar	AIC	ΔAIC	AIC Weight
	(a) *ψ*_S:W_				
	(p = age * sex + year + nr:adult + a1:mtHap3 + weanW:juv)				
	*(* *Φ* = sex*age + nr + a0:mtHap3:regN + a1:weanW)				
10	a01:sex + a2 + a3p	69	966.5	0.0	0.28
6	a01:sex + a2:sex + a3p	70	967.0	0.5	0.22
4	a01 + a2 + a3p	68	967.5	1.0	0.17
11	a01:reg + a2 + a3p	69	969.4	2.9	0.07
1	a0 + a1 + a2 + a3p	69	969.5	3.0	0.06
9	a01 + a2 + a3p + a01:mtW + a2:mtW	70	969.6	3.0	0.06
13	a01:reg:mtHap2 + a2 + a3p	70	970.0	3.5	0.05
3	a0:sex + a1:sex + a2:sex + a3p	72	970.7	4.2	0.03
5	a01:reg + a2:reg + a3p	70	971.4	4.9	0.02
12	a01:reg:mtHap3 + a2 + a3p	71	971.9	5.4	0.02
2	a0:reg + a1:reg + a2:reg + a3p	72	973.4	6.9	0.01
8	a01:reg:mtHap2 + a2:reg:mtHap2 + a3p	72	973.9	7.4	0.01
7	a01:reg:mtHap3 + a2:reg:mtHap3 + a3p	74	975.9	9.4	0.00
	(b) *p*				
10	age * sex + year +nr:adult + a1:mtHap3 + weanW:juv	69	966.5	0.0	
14	age * sex + year + nr:adult + a1:mtHap3	68	985.2	18.7	
	(c) Φ				
18	base + a1:weanW:regS	68	964.9	0.0	0.20
24	base + a12:weanW:regS	68	965.2	0.3	0.17
16	base + a1:weanW	68	966.1	1.2	0.11
22	base + a1:weanW:regS:sex	69	966.5	1.6	0.09
10	base + a1:weanW:reg	69	966.5	1.6	0.09
23	base + a1:weanW:regS + a2:weanW:regS	69	966.6	1.7	0.08
19	base + a1:weanW:sex	69	967.0	2.1	0.07
15	sex*age + nr + a0:mtHap3:regN (base)	67	967.1	2.2	0.06
20	base + a1:weanW:regS + a1:weanW:mtHap2	70	967.8	2.9	0.05
17	base + a12:weanW	68	967.9	3.0	0.04
21	base + a1:weanW:regS + a1:weanW:mtHap3	71	968.8	3.9	0.03
	(d) Φ, a0:mass				
26	Best + a0:bs(mass)	72	947.3	0.0	0.64
27	Best + a0:bs(mass, *df* = 4)	73	949.7	2.4	0.20
28	Best + a0:bs(mass, *df* = 5)	74	949.6	4.2	0.08
29	Best + a0:bs(mass, *df* = 6)	75	949.8	4.5	0.07
25	Best + a0:mass	70	954.4	8.6	0.01
10	Best *(=base + a1:weanW:reg)*	69	966.5	19.2	0.00
	(e) Φ, a1:weanW:mass				
26	Best + a0:bs(mass)	72	947.3	0.0	0.70
30	Best + a0:bs(mass) + a1:weanW:mass	73	949.4	2.1	0.25
31	Best + a0:bs(mass) + a1:weanW:bs(mass)	75	952.7	5.4	0.05
	(f) *ψ*_S:W_, a0:mass				
	*(* Φ * = model 26)*				
32	Best + a0:mass	73	940.4	0.0	0.80
33	Best + a0:bs(mass)	75	943.5	3.1	0.17
26	Best *(=a01:sex + a2 + a3p)*	72	947.3	6.9	0.03

Note

weanW = weaned, nr = natal rookery, reg = regions: N = born "north" (Graves Rocks/White Sisters), S = born "south" (Forrester/Hazy Islands), mtHap = maternal haplotype for pups born at Graves Rocks and White Sisters: mtHap2 = known/unknown haplotype, mtHap3 = eastern/western/unknown haplotype, fem = females, mal = males, a0/1/2/3 = age 0/1/2/3. a01 = common parameters fit for a0 and a1. *ψ*_S:W_ = weaning probability, *p* = animal detection probability, *Φ* = survival probability. bs = mass effect fit as *b*‐spline smooth. Mass was deviation from the sex‐specific means of neonatal body mass (adjusted for capture date/age) in kg, included as an individual covariate.

The best age structure for weaning probability (*ψ*_S:W_) had a common probability of weaning from ages 0 to 1 and 1 to 2, with *ψ*_S:W_ higher at ages 2–3 and ~1.0 at ages 3+ (Table 4; Table 3a: model 1 vs. 4, ~3x AIC Weight). When estimated separately, $\hat{\psi }$_S:W_ were essentially equivalent: 0.587 *[0.499, 0.670]* and 0.568 *[0.435, 0.693]* at ages 0–1 and 1–2, respectively (model 1 in Table 3a). The top ranked *ψ* models included a sex effect for *ψ*_S:W_ at ages 0–2 due to a 0.08 higher weaning probability for females than for males (Table 4). Although AIC Weight supported this sex effect, definitive statistical support was weak as ΔAIC was only 1.0 (Table 3a: model 10 vs. 4). The sex effect for *ψ*_S:W_ was retained when modeling *Φ*. Regional or *mtHap* variation in *ψ* was not statistically supported (Table 3a). Point estimates (ages 0–2) were nearly identical for weaning probabilities in the north and south regions (0.587 vs. 0.572, model 11 in Table 3a), and for *mtW* and *mtE* animals from the north (0.561 vs. 0.531, model 12 in Table 3a).

**Table 4:** Weaning probabilities (*ψ*_S:W_) of Steller sea lions in Southeast Alaska (SEAK) and the Gulf of Alaska (GOA) by natal rookery, sex, and age

Group		*ψ*_S:W_	95% CI
SEAK	a01, females	0.616	*[0.538, 0.690]*
SEAK	a01, males	0.536	*[0.441, 0.627]*
SEAK	a2	0.905	*[0.764, 0.966]*
SEAK	a3p	1.000	*[1.000, 1.000]*
Ugamak, GOA	a0*	0.836	*[0.778, 0.881]*
PWS, GOA	a0*	0.601	*[0.246, 0.874]*
Marmot, GOA	a0*	0.676	*[0.598, 0.744]*
Sugarloaf, GOA	a0*	0.681	*[0.593, 0.757]*
Ugamak, GOA	a1*	0.512	*[0.287, 0.732]*
PWS, GOA	a1*	0.591	*[0.176, 0.907]*
Marmot, GOA	a1*	0.698	*[0.492, 0.846]*
Sugarloaf, GOA	a1*	0.186	*[0.041, 0.551]*
all GOA	a01summer	0.118	*[0.046, 0.269]*
all GOA	a2p	0.720	*[0.493, 0.872]*

Note

PWS = Prince William Sound. Ages for SEAK were a0 (ages 0–1), a1 (ages 1–2), a2 (ages 2–3), a3p (annual rate, age > 2), and a01 (rate at a0 and a1 was equal), where the seasonal cutoff time for weaning was 20 June. Ages for GOA were a0* (age 0–age 1 early season), a1* (age 1 late season–age 2 early season), a2p (annual rate > age 2 late season), and a01summer (early season to late season equal at ages 1 and 2), where early season cutoff was 1 May–19 June and the late season cutoff was as in the SEAK analysis, 20 June. Estimates for SEAK are from model 10 in Table 3 and for GOA were from model 21 in Table 5.

**Figure 4:**
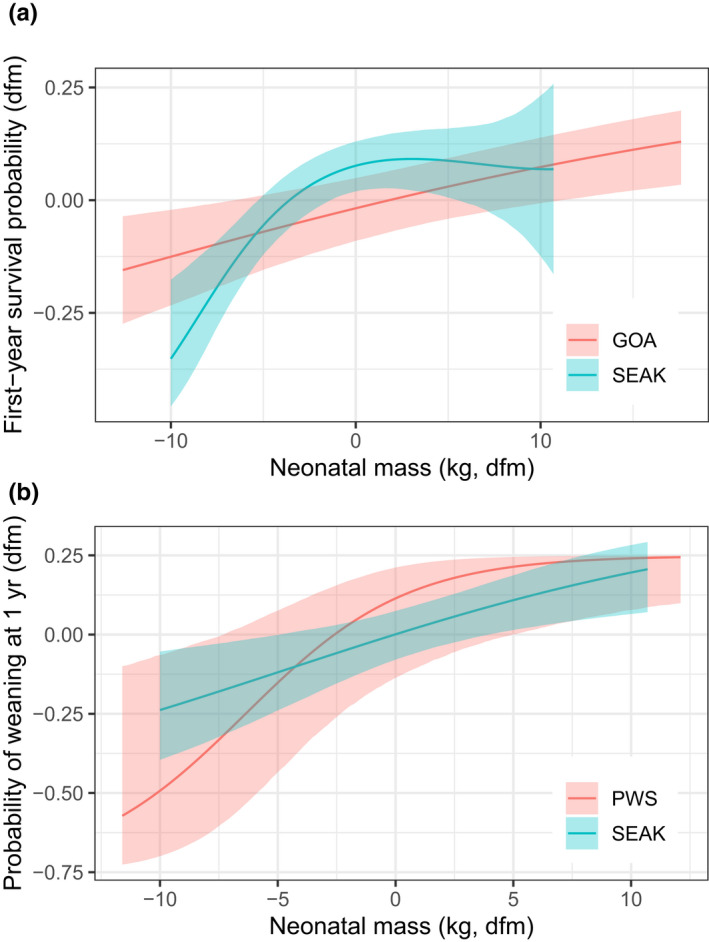
Effect of neonatal body mass on first‐year survival probability (a) and probability of weaning at age 1 (b) for Steller sea lions born in Southeast Alaska (SEAK) and the Gulf of Alaska (GOA, see caption for Figure 1). Results are presented as deviations from the group‐specific means (dfm) for both survival or weaning probabilities (response variables) and neonatal mass (predictor variable). Data are plotted for Forrester Island Complex females (SEAK) and Ugamak Island females (GOA) in (a) and for Forrester Island Complex females and Prince William Sound females (PWS, including Fish Island and Seal Rocks) in (b). Ribbons are 95% CI

For survival, $\hat{\Phi }$ from age 1 to 2 was lower for weaned than unweaned yearlings, and this survival effect was well supported for animals born at southern rookeries (model 18 vs. 15 in Table 3c, ΔAIC = 2.2, ~3x AIC Weight). The effect of weaning on survival averaged −0.183 and −0.049 for yearlings from the south and north, respectively (model 10 in Table 3c). The effects of weaning on survival did not differ by sex or *mtHap* (Table 3c). As expected (Hastings et al., 2011), first‐year survival was positively related to neonatal body mass, but the functional form of this relationship (best fit with the *b‐*spline smooth) was nonlinear rather than the linear pattern previously reported (Table 3d, Figure 4a). No effect of neonatal body mass on survival of weaned yearlings was supported (Table 3e), but probability of weaning at age 1 increased with neonatal size in a linear manner (Table 3f, Figure 4b).

### 3.3 Gulf of Alaska

**Figure 5:**
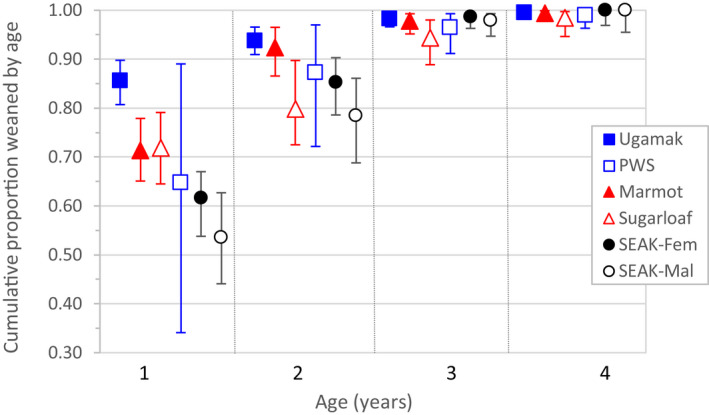
Cumulative proportion of Steller sea lions weaned by 1–4 years of age by natal rookery and sex. Error bars are 95% CI. See Table 6 for estimates. Six rookery * sex groups were Ugamak Island, Marmot Island, Sugarloaf Island, Prince William Sound (PWS), and males and females in Southeast Alaska (SEAK). Sex differences were apparent only in SEAK; weaning probabilities did not vary with natal rookery in SEAK. Cumulative values were calculated using estimates of weaning probabilities ($\hat{\psi }$_S:W_) from model 10 in Table 3 and model 21 in Table 5

The average number of branded juveniles resighted per year in the Gulf of Alaska during the EARLY and LATE periods, respectively, was 71 (range: 11–180 among years) and 78 (range: 25–125). When the most definitive behavior was summarized per summer per animal, 292 summer * animals were best observed as "S": 165/73 at age 1 EARLY/LATE seasons (57%/25%), 34/15 at age 2 EARLY/LATE (11%/5%), 5 at age 3 EARLY (2%), and 0 after age 3 EARLY. Probability of detecting the weaning status of an unweaned juvenile per observation ($\hat{\delta }$) was very similar to that from the Southeast Alaska analysis at 0.253 *[0.226, 0.283]*. A single, complex *p* model was strongly preferred (AIC Weight = 0.76) which included multiplicative effects for nearly all variables; *p* of weaned versus unweaned juveniles also varied in a complex manner (Table 5a). Unlike the Southeast Alaska analyses, separate *ψ*_S:W_ for a0 and a1 was supported along with effects of natal rookery (Table 5b). Natal rookery rather than "region" was preferred due to a particularly low weaning probability at a1 for Sugarloaf Island animals but not for Marmot Island animals (Table 4; model 12 vs. 11 in Table 5b); the estimates for Prince William Sound were imprecise due to small sample size (Table 4). No sex differences in weaning probabilities were supported: When a sex effect was included, point estimates of sex‐specific probabilities were very similar (0.730 and 0.739 at a0, 0.621 and 0.590 at a1, for females and males, respectively). Based on the estimates of the age‐specific cumulative proportion weaned, Ugamak Island animals weaned at the youngest ages and central Gulf animals at the next youngest ages (Figure 5, Table 6). Prince William Sound animals weaned at slightly older ages than the other Gulf of Alaska rookeries and estimates of age‐specific cumulative proportion weaned for Prince William Sound animals were similar to those for females from Southeast Alaska (Figure 5). Probabilities were lowest for Southeast Alaska males at ages 1–2 and for Sugarloaf Island animals at age 2 (Figure 5).

**Table 5:** Model selection results for Gulf of Alaska analysis

Model#	Model	Npar	AIC	ΔAIC	AIC Weight
	(a) *p*				
	*(ψ*_S:W_ = *a0 + a1 + a01summer + a2 + a3p)*				
	*(* Φ = sex*ac4*nr + weanW:a1:nr)				
8	ac2 * reg * survey * weanW:juv + sex * ac2	254	485.0	0.0	0.76
6	ac2 * reg * survey * weanW:juv + sex	253	487.4	2.3	0.24
7	ac2 * reg * survey + sex + weanW:juv	188	559.4	74.4	0.00
4	ac2 * reg * survey + sex	187	616.0	131.0	0.00
1	ac2 * sex * reg * survey	326	727.4	242.4	0.00
5	sex * reg * survey + ac2	231	925.6	440.5	0.00
2	sex * reg * survey	138	1,266.4	781.3	0.00
3	reg * survey	230	1,310.5	825.5	0.00
	(b) *ψ*_S:W_				
11	a0:nr + a1:nr + a01summer + a2p	259	469.7	0.0	0.66
14	a0:nr + a1 + a01summer + a2p	256	471.7	2.0	0.24
12	a0:reg + a1:reg + a01summer + a2p	257	474.1	4.4	0.07
15	a0:nr:sex + a1:nr + a01summer + a2p	263	476.7	7.0	0.02
9	a0 + a1 + a01summer + a2p	253	483.0	13.3	0.00
10	a01 + a01summer + a2p	252	484.3	14.6	0.00
8	a0 + a1 + a01summer + a2 + a3p	254	485.0	15.4	0.00
13	a0:sex + a1:sex + a01summer + a2p	255	486.9	17.2	0.00
	(c) *Φ*				
21	sex * ac4 * nr + weanW:a1 = a2:CGOA	256	456.9	0.0	0.45
25	sex * ac4 * nr + weanW:a1 = a2:CGOA + weanW:a1:other	257	458.1	1.2	0.25
24	Model 21 + weanW:a1:Ugamak + weanW:a1:PWS	258	459.9	3.0	0.10
23	sex * ac4 * nr + weanW:a1 = a2:reg	258	460.1	3.2	0.09
18	sex * ac4 * nr + weanW:a1 = a2	256	460.8	3.9	0.06
19	sex * ac4 * nr + weanW:a1 = a2:nr	259	462.0	5.1	0.04
20	sex * ac4 * nr + weanW:a1:CGOA	256	464.9	8.0	0.01
17	sex * ac4 * nr + weanW:a1	256	466.4	9.5	0.00
22	sex * ac4 * nr + weanW:a1:reg	258	468.0	11.1	0.00
11	sex * ac4 * nr + weanW:a1:nr	259	469.7	12.8	0.00
16	sex * ac4 * nr	255	475.8	19.0	0.00
	(d) Φ, a0:mass				
26	Best + a0:mass	257	447.2	0.0	0.56
30	Best + a0:mass:sex	258	449.2	2.0	0.21
27	Best + a0:bs(mass)	259	450.5	3.3	0.11
29	Best + a0:mass:nr	260	451.0	3.8	0.08
28	Best + a0:mass:reg	259	452.9	5.7	0.03
21	(Best)	256	456.9	9.7	0.01
	(e) Φ *,* a1:weanW:mass				
26	Best + a0:mass	257	447.2	0.0	
31	Best + a0:mass + a1:weanW:mass	258	449.1	1.9	
	(f) *ψ*_S:W_, a0:mass				
36	Best + a0:mass:J * *post‐hoc model*	258	438.9	0.0	0.79
35	Best + a0:mass:reg	260	442.4	3.5	0.14
34	Best + a0:mass:nr	261	444.3	5.4	0.05
26	(Best)	257	447.2	8.3	0.01
32	Best + a0:mass	258	448.8	9.9	0.01
33	Best + a0:bs(mass)	260	449.9	11.0	0.00

Note

ac2 = age class (juveniles 1–3, adult 4+), ac4 = age class (0, 1, 2, 3+[females] or 3–8 and 9+ [males]), reg = region (Central Gulf: CGOA, Prince William Sound: PWS, Ugamak, see Figure 1), weanW = weaned, juv = juvenile, a0/1/2/2p/3p = age 0/1/2/2+/3+, summer = interval between early (May–20 June) and late (20 June–August). a01 = common parameters fit for a0 and a1. *ψ*_S:W_ = weaning probability, *p* = animal detection probability, Φ = survival probability. bs = mass effect fit as *b*‐spline smooth. Mass was deviation from the sex‐specific means of neonatal body mass (adjusted for capture date/age) in kg, included as an individual covariate.

**Table 6:** Proportion of Steller sea lions weaned by age, natal rookery, and sex *[95% CI]*

Natal rookery	Age (years)			
	1	2	3	4
Ugamak	0.855 *[0.807,0.898]*	0.938 *[0.909,0.966]*	0.983 *[0.966,0.993]*	0.995 *[0.984,0.999]*
Marmot	0.714 *[0.651,0.779]*	0.924 *[0.866,0.965]*	0.979 *[0.952,0.993]*	0.994 *[0.978,0.999]*
Sugarloaf	0.718 *[0.645,0.791]*	0.798 *[0.725,0.897]*	0.943 *[0.889,0.980]*	0.984 *[0.946,0.997]*
PWS	0.648 *[0.341,0.890]*	0.873 *[0.722,0.970]*	0.964 *[0.912,0.993]*	0.990 *[0.963,0.999]*
SEAK, females	0.616 *[0.538,0.670]*	0.853 *[0.786,0.903]*	0.986 *[0.963,0.995]*	1.000 *[0.969,1.000]*
SEAK, males	0.536 *[0.441,0.627]*	0.784 *[0.688,0.861]*	0.980 *[0.947,0.993]*	1.000 *[0.955,1.000]*

Note

Values were derived from parameter estimates in model 10 in Table 3 for Southeast Alaska (SEAK) animals and model 21 in Table 5 for Gulf of Alaska animals. PWS = Prince William Sound.

The effects of weaning on survival occurred at ages 1 and 2 and were severe for central Gulf animals (Table 5c). Survival of these animals was reduced due to weaning by an absolute value of −0.215 to −0.296 at age 1 and −0.135 to −0.187 at age 2 (model 25 in Table 5c). Although not supported, point estimates of the reduction in survival attributable to weaning were −0.078 to −0.093 for Ugamak Island yearlings and were −0.015 to −0.038 for Prince William Sound yearlings (model 24 in Table 5c). First‐year survival was positively related to neonatal body mass, but unlike the Southeast Alaska pattern, the relationship was linear (Figure 4a, Table 5d). As in Southeast Alaska, survival of weaned yearlings was unrelated to neonatal body mass (Table 5e), but in contrast to the Southeast Alaska results, the probability of weaning at age 1 was unrelated to neonatal size for most rookeries in the Gulf of Alaska (model 26 vs. 32/33 in Table 5f). A regional difference in the effect of neonatal size on weaning rate at age 1 was supported (model 35 vs. 26 in Table 5f), due to an unexpectedly large positive effect for Prince William Sound animals ($\hat{\beta }=0.293$ *[0.070, 0.515]*) in contrast to no supported effect for other regions ($\hat{\beta }$[central Gulf] = −0.011 *[−0.053, 0.032]*; $\hat{\beta }$ [Ugamak] = −0.016 *[−0.083, 0.050]*; model 35 in Table 5f). The relationship for Prince William Sound animals was poorly estimated, and the large effect size may be a spurious result for this small sample (Figure 4b). Model selection results and parameter estimates were essentially identical whether body mass was adjusted or unadjusted for capture date/age before inclusion in models in either dataset.

**Figure 6:**
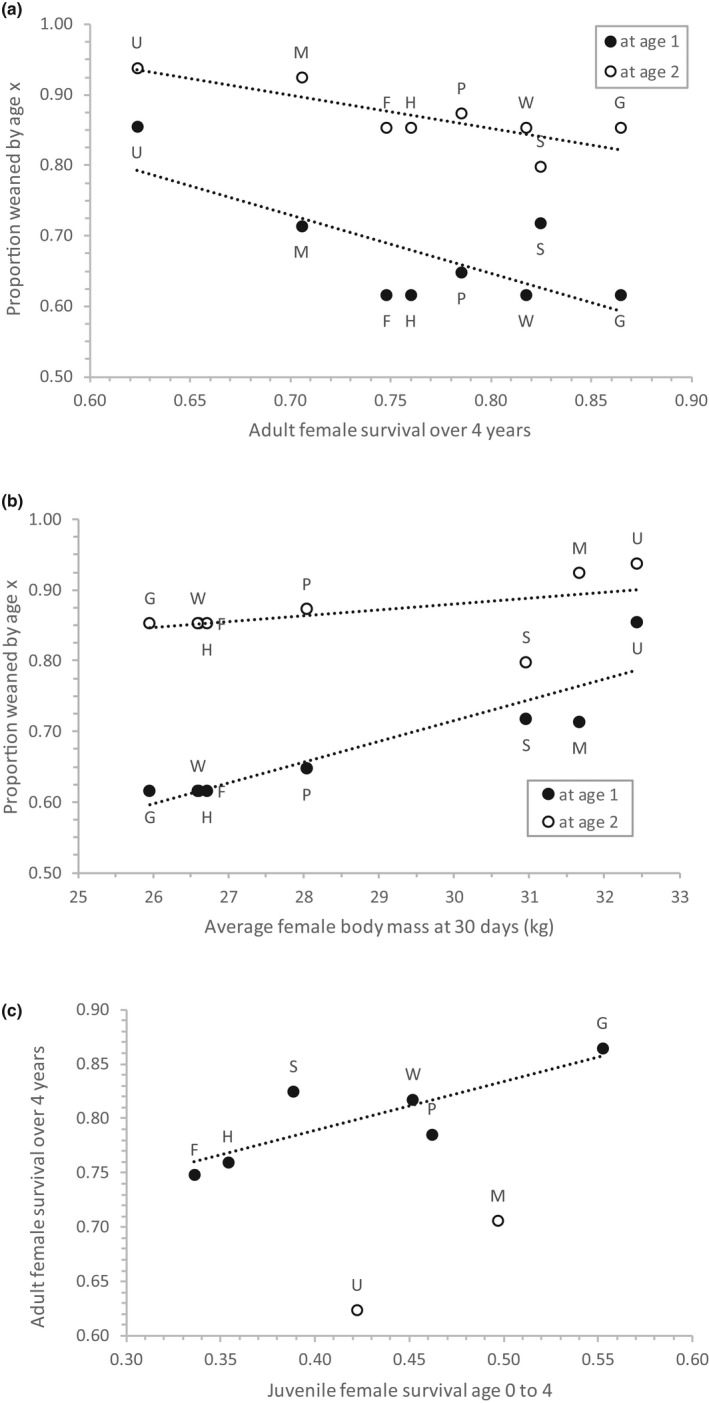
Relationships between the cumulative proportions of female pups weaned by age 1 or 2 and adult female survival probability (a) or neonatal body mass of females (b); and between juvenile and adult survival of females (c). Data labels show rookery‐specific values: U = Ugamak Island, M = Marmot Island, S = Sugarloaf Island, P = Prince William Sound, G = Graves Rocks, W = White Sisters, H = Hazy Islands, and F = Forrester Island Complex. Neonatal body mass (b) is mean estimated body mass of females at 30 days after the rookery‐specific mean birth date. Juvenile survival (c) is survival of females from 0 to 4 years of age, and adult survival is survival of females over a 4‐year period (e.g., ages 4–8). In (c), U and M were outliers not included in the trend line shown

Finally, adult female survival was lower at rookeries where juveniles were weaned at a younger age and where neonates were larger (all *p* < 0.001, Figure 6a–b), but not strongly correlated with regional or rookery‐specific trends. Although our sample of rookeries was small (*n* = 9 pooled into 8), for most rookeries, juvenile female survival was 45%–64% of adult female survival, and as juvenile survival increased so did adult female survival (*p* < 0.001 for six rookeries, Figure 6c). But for the two rookeries with highest proportion weaned by age 2 (Marmot and Ugamak), juvenile female survival was in the mid‐range of observed values, but adult female survival was low (Figure 6c). Variation in the effect of weaning on survival was not strongly correlated with any population parameters.

## 4 DISCUSSION

The key findings of our study include (a) precise estimates of age‐specific weaning probabilities for Steller sea lions across a large portion of their range demonstrating that delayed weaning beyond 1 year was common (up to 46% and 22% of juveniles continued to suckle in their second and third years, respectively, in some areas); (b) age‐specific weaning patterns varied among sexes only in Southeast Alaska, where females weaned slightly earlier than males; (c) spatial variability in weaning age mirrored spatial variability in animal body size (i.e., weaning probability averaged 0.30 higher at age 1 for the larger animals at western rookeries [endangered population] compared with the smaller animals at rookeries in the eastern Gulf of Alaska and Southeast Alaska); (d) spatial variability in weaning age was unrelated to population trends but earlier weaning and larger offspring size were associated with reduced adult female survival; and (e) the effect of weaning on survival of juveniles may be an important metric to indicate areas with poor conditions for sea lions: Weaning resulted in significantly reduced survival of weaned versus unweaned juveniles in the central Gulf and southern Southeast Alaska but not other areas.

Steller sea lions in Alaska regularly delayed weaning their offspring for >1 year, with most variability at age 1, when 0.54–0.86 were weaned, compared with 0.78–0.94 weaned at age 2. Most individuals had weaned by age 3 (0.94–0.99, Figure 5, Table 6). Variability in this trait suggests that flexibility in the length of the offspring dependency period is an important aspect of life history and fitness in this species. Besides the two most polar fur seal species with a particularly short lactation period of 4 months (northern fur seals *Callorhinus ursinus* and Antarctic fur seals *Arctocephalus gazella*, Gentry & Holt, 1986, Doidge & Croxall, 1989), delayed weaning beyond age 1 has been documented in half of the 12 other otariid species (Galapagos fur seals *Arctocephalus galapagoensis*, Galapagos sea lions *Zalophus wollebaeki,* Australian sea lions *Neophoca cinerea,* South American fur seals *Arctocephalus australis*, and possibly southern sea lions *Otaria flavescens* and the two subspecies of the brown fur seal *Arctocephalus pusillus doriferus* and *Arctocephalus pusillus pusillus*; David & Rand, 1986, Hamilton, 1934, Higgins & Gass, 1993, Hume et al., 2001, Lowther & Goldsworthy, 2016, Rand, 1955, Trillmich & Majluf, 1981, Trillmich & Wolf, 2008, Vaz‐Ferreira, 1979). In contrast, this ability has not been documented in the other six species which usually wean at 9–11 months (Chilvers et al., 2007; Francis et al., 1998; Gallo‐Reynoso & Figueroa‐Carranza, 2010; Georges & Guinet, 2000; Haase, 2004; Harris, 2016; Melin et al., 2000).

That many otariid species demonstrate the ability to delay weaning past age 1 suggests this ability is important to fitness of otariids in general but causes for interspecific variation are not well understood. It is suspected to especially occur in species at low latitudes and in areas where food supply is particularly patchy, poor, or is unpredictably and highly variable (Gentry & Kooyman, 1986; Lowther & Goldsworthy, 2016; Trillmich et al., 1991). However, it is observed in both tropical and subpolar otariids; some species strongly affected by unpredictable annual variability in food supply do not demonstrate this ability (e.g., California sea lions during El Niño); and its' association with poor food conditions in those otariid species demonstrating this ability is also not well‐documented (but see Jeglinski et al., 2012; Trillmich, 1986). For this question, an interspecific comparison of energetics (e.g., of milk production, metabolism, thermoregulation, foraging, and diet) involved in producing offspring of adequate weaning size for survival (Lee et al., 1991), and possibly genetic constraints, is needed.

Although later weaning in males may be expected, as higher investment in male than female offspring to weaning has been observed in many polygynous and sexually dimorphic mammals (Clutton‐Brock et al., 1981), only weak evidence suggested later weaning of male than female Steller sea lions and only in Southeast Alaska (a finding similar to that of Trites et al., 2006). Weaning ages did not vary by sex in the Gulf of Alaska (although sample sizes from Prince William Sound were small) and survival effect of early weaning did not vary with sex in any region. At Chiswell Island, weaning age was also similar between sexes, but survival costs of early weaning were ~0.20 higher for males than females (Figure 3 in Maniscalco, 2014).

### 4.1 Spatial variability and the importance of offspring size to fitness

Weaning ages were similar for animals born at the easternmost areas, Southeast Alaska and Prince William Sound, and were progressively earlier for those born to the west (Figure 5). This result is consistent with data from isotopic signatures in whiskers (L. Rea *unpublished data*) and with time‐at‐sea data from instrumented juveniles (Call et al., 2007), which suggested earlier weaning for juveniles in the Aleutian Islands and/or central Gulf of Alaska compared with Prince William Sound and Southeast Alaska. "Gradual" (where offspring supplement mother's milk with independent foraging) versus "abrupt" weaning cannot be distinguished in our study; gradual weaning at the easternmost areas versus abrupt weaning to the west may be an aspect of the observed spatial patterns. Gradual weaning has been documented in several otariid species (David & Rand, 1986; Horning & Trillmich, 1997; Jeglinski et al., 2012; Lowther & Goldsworthy, 2016) and may also occur in Steller sea lions in Southeast Alaska (L. Rea *unpublished data*). Whether gradual or abrupt, later weaning either directly reduces or reflects reduced reproductive output of females in the eastern areas, because females are only rarely observed with two dependent offspring and very rarely observed nursing two dependent offspring simultaneously through the entire breeding season (Maniscalco & Parker, 2009).

Spatial variation in weaning ages did not reflect population trends, as would be expected if both population trend and this life‐history trait were simply responsive to food conditions (e.g., later or earlier weaning was associated with poorer food conditions in areas of slower population growth). This was most apparent in Southeast Alaska where weaning ages were nearly identical, yet the population in the south has a stable long‐term population trend, low survival rates, and large animal geographic ranges, and in the north has high population growth, the highest survival rates observed range wide and contracted animal ranges (Hastings et al., 2011; Jemison et al., 2018; Mathews et al., 2011; Wright et al., 2017). This suggests different suites of life‐history traits may have similar fitness in this species. Similarly, individual Swedish brown bears (*Ursus arctos*) also demonstrated either short (~1.5 years) or long (~2.5 years) periods of maternal care, and the two tactics, together with associated trade‐offs in survival and reproduction, produced similar fitness (Van de Walle et al., 2018).

Instead, spatial variation in weaning age and body size was most similar, with body sizes larger in the west where weaning was earlier. Larger body size of neonates, juveniles, and adults in the west compared with Southeast Alaska and Prince William Sound has been previously reported for this species (Brandon et al., 2005, Merrick et al., 1995, Rea et al., 2016, Sweeney et al., 2015 [see Figure 3 in Sweeney et al, 2015 for largely nonoverlapping CI for adults in Prince William Sound versus western sites]) and also in harbor seals *Phoca vitulina* in these same areas (seals from Southeast Alaska and Prince William Sound were 12%–23% lighter as fetuses, newborns, and adults than those from the central Gulf; Pitcher & Calkins, 1979).

The large size of westernmost sea lion pups was likely due to high neonatal growth rather than greater birth weights (Brandon et al., 2005). A suspected cause of high early growth in the west was greater female attendance and presumably milk transfer due to short maternal foraging trip durations during the breeding season (Andrews et al., 2002; Brandon et al., 2005; Milette & Trites, 2003), where prey may have been more abundant (Andrews et al., 2002), concentrated (Winter et al., 2009), predictable, or closer to rookeries in summer. This hypothesis requires further study: A larger dataset suggested maternal foraging trip duration may be more locally influenced than implied by previous study results (Figure 5–15 in National Marine Fisheries Service, 2014). During poor food conditions, the adoption of maternal strategies that reduce time fasting and conserve consistent maternal attendance of offspring may be critical to resilience of sea lion populations (McHuron et al., 2017a; Melin et al., 2000).

A negative relationship between offspring growth rates and time to weaning is expected for large mammals, and the threshold body size commonly observed (at ~four times birth weight; Lee, 1996; Schulz & Bowen, 2005) may result from the inability of mothers to meet offspring energy needs through lactation once offspring reach a certain size (Lee et al., 1991). The faster growth of westernmost Steller sea lion pups may have allowed them to reach the threshold weaning weight faster than easternmost pups resulting in earlier weaning ages. Weaning masses (currently unknown), rather than neonatal masses, would particularly inform this hypothesis. However, the probability of weaning at age 1 was dependent on neonatal body mass in Southeast Alaska but not in the Gulf of Alaska (Figure 4b), suggesting that body size may determine time to weaning for sea lions. The lack of pattern in the west may suggest most neonates were large enough and perhaps grew fast enough to reach threshold size within the first year. Similarly, whether Swedish brown bears weaned their offspring at either ~1.5 or ~2.5 years depended on yearling body size and support of offspring through an additional year compensated for low yearling mass (Dahle & Swenson, 2003).

The larger size of westernmost versus easternmost animals throughout the first year (Rea et al., 2016) and as adults (Sweeney et al., 2015) suggests high growth in the first year translates to larger size when older and/or that selection for large size is stronger in the west than the east throughout the lifespan. A larger optimal offspring body size in the west than east is supported by a positive linear relationship in first‐year survival with offspring body size; survival was highest for the largest neonates (Figure 4a). In contrast, first‐year survival did not improve with neonatal body size for sea lions in Southeast Alaska above the average size but declined sharply below the average (Figure 4a). Therefore, being larger than average may not benefit offspring survival in Southeast Alaska, although being at least average size was critical to their survival. Prince William Sound sea lions may have a similar nonlinear pattern, given their other similarities to Southeast Alaska sea lions, but small sample sizes prevent a thorough investigation. Unlike at Chiswell Island where birth mass greatly affected survival of weaned yearling Steller sea lions (Δ 0.40 absolute value; Maniscalco, 2014), neonatal size affected first‐year survival only and not weaned yearling survival in our study, but yearling body size would better inform this analysis.

Environmental factors underlying spatial patterns require more study, but geographic variation in habitat, prey availability, and diet suggests potential links. The nearshore environment is important to juveniles and adult females (Merrick & Loughlin, 1997; Raum‐Suryan et al., 2004; Sinclair & Zeppelin, 2002). Prince William Sound and Southeast Alaska are characterized by complex coastlines, narrow continental shelves (as narrow as < 15 km), and deep water nearshore (up to ≥700 m), and are similar in fish composition (Mueter & Norcross, 2002). The continental shelf is broad and shallow (usually < 100 m and rarely > 250 m) around Sugarloaf and Marmot Islands, and at Ugamak Island, the deeper waters nearshore are oceanographically dynamic (characterized by spatial heterogeneity and temporal stability; Lander et al., 2009) and bathymetrically diverse, with patches of unusually high prey biomass and species richness (reviewed by Fadely et al., 2005).

Habitat differences may translate to broad‐scale variation in prey populations but fish studies are limited, especially of Steller sea lion prey in the nearshore. The waters west of Prince William Sound were much higher in fish abundance and lower in fish diversity than those in Prince William Sound and Southeast Alaska using data from a multidecadal summer bottom trawl survey (Mueter & Norcross, 2002). Similarly, although diet studies in Prince William Sound are lacking, greater fish diversity occurred in sea lion diets in Southeast Alaska compared with western sites, and the dominant prey in both areas (walleye pollock *Gadus chalcogrammus*) was more often consumed with other energy‐rich prey in Southeast Alaska (Trites et al., 2007). Adult females dive deeper in Southeast Alaska than in the west (Lander et al., 2020); sea lions in the easternmost areas may require more diverse and flexible foraging strategies to access diverse or more dispersed prey in deep waters. If so, later weaning may allow mothers time to teach foraging areas and skills, as suspected for Australian sea lions (Lowther & Goldsworthy, 2016 and references therein) and Odonocetes (reviewed by Matthews & Ferguson, 2015). Despite potential patterns, the complex relationships between habitat, diet, and sea lion life history remain largely unknown, including the importance to sea lion energetics of diet diversity (Fritz et al., 2019; Lander et al., 2009; Merrick et al., 1997), local variation in prey abundance (e.g., prey conditions for sea lions in northern Southeast Alaska are likely very favorable and are fueling high survival and population growth; Hastings et al., 2011; Mathews et al., 2011), and prey concentration (Rand et al., 2019; Winter et al., 2009), predictability (Baylis et al., 2012), and seasonality (Varpe, 2017). As yet, distinct regional differences in foraging effort or strategies have not been detected (Lander et al., 2020; Loughlin et al., 2003; Merrick & Loughlin, 1997; Pitcher et al., 2005; Raum‐Suryan et al., 2004; Rehberg et al., 2009).

### 4.2 Maternal effects, trade‐offs among life‐history traits, and management implications

Maternal body size may be an important aspect of the spatial patterns we observed. Due to smaller body size of mothers in Southeast Alaska (and possibly Prince William Sound, see Figure 3 in Sweeney et al., 2015), mothers in these regions may operate closer to the edge of their biological capacity for supporting dependent offspring through late lactation with the potential for higher late‐term abortion rates, particularly during poor prey conditions (Pitcher et al., 1998). Body condition when lactating late in gestation (January–May) determines abortion rates in Steller sea lions, which can range to >30% (Pitcher et al., 1998). Eastern females that abort their fetus may, if they are physically capable, sustain their dependent juveniles even partially, through another year, and improve their fitness. Therefore, if low birth rates also occur in Southeast Alaska and Prince William Sound (currently under study), longer lactation periods may be a consequence of higher abortion rates.

Larger maternal body size may also be more optimal in the west, such as would result if optimal reproductive performance is more dependent on maternal condition in the west than the east (McNamara & Houston, 1996). Reproductive performance of subantarctic fur seals *Arctocephalus tropicalis* depended greatly on maternal quality and body size (Beauplet & Guinet, 2007), perhaps by improving the probability that pups endured and survived long fasts during late lactation and postweaning (Verrier et al., 2011). Therefore, large body size may be selected for in environments that experience challenging food conditions, even for a period of their lives (Verrier et al., 2011). During the years of the 1976/1977 regime shift and presumed rapid change in prey populations, only large pups that weaned earlier survived in the central and eastern Gulf (York et al., 2008). If large body size particular aids female fitness in the west, larger or older females may produce more offspring and/or mothers may invest heavily in offspring with higher costs of reproduction (e.g., in terms of longevity or reproductive lifespan). Negative correlation between adult female survival and probability of offspring weaning by ages 1 and 2 at Marmot and Ugamak where weaning ages were youngest suggests that females at these sites may trade‐off their survival for production of offspring with high initial growth. A significant portion of western females may also be nonreproductive if they are not of sufficient quality to produce large, viable offspring. Large size may also be selected for in populations if early reproduction is strongly selected for, as sexual development is more related to body weight than to age in mammals (Widdowson, 1981).

Western mothers may benefit from incorporating more capital into provisioning their offspring than eastern mothers due to adaptation to different long‐term average conditions in different habitats. An increased reliance on stored energy for offspring provisioning may be favored in areas with increased food availability, seasonality, and unpredictability (particularly very seasonally abundant food supply, Stephens et al., 2014; Varpe, 2017). The ability to store energy scales linearly with body mass (Prothero, 1995) and seasonality in prey can be a driver of large body size (Lindstedt & Boyce, 1985). The degree of use of capital may be plastic within a species depending on food conditions and the degree of seasonality (reviewed by Williams et al., 2017). The limited existing evidence suggests that pups were larger in the west than in the east before, during, and after the decline (Brandon et al., 2005, Merrick et al., 1995, B. Fadely unpublished analyses), perhaps reflecting different long‐term average conditions in these habitats. A suite of life‐history traits dependent on greater use of capital to fuel higher offspring growth and large body size may be particularly risky to otariids, if they lack plasticity to shift tactics during rapid and severe shifts in conditions (Forcada et al., 2008). The two largest bodied otariids (Steller and southern sea lions) have historically large populations concentrated at high latitudes where prey is abundant, predictable, and highly seasonal. A similarly severe population crash coincident with ocean warming was observed for southern sea lions at the Falkland Islands where numbers declined by 95% (from >350,000 animals to <30,000) over a ~30‐year period (the 1930s–1965) and have not recovered (Baylis et al., 2015).

Finally, our study suggests that the effect of weaning on survival (Δ in *Φ* for weaned vs. unweaned juveniles) may indicate poor areas for sea lions (e.g., areas of poor food/higher predation or fisheries interactions) and provide a useful metric for population monitoring. This metric did not directly correlate with population trends, but particularly reduced survival due to weaning occurred in southern Southeast Alaska (−0.18 absolute value, where the population is at carrying capacity) and in the central Gulf (−0.25 and −0.15 ages 1 and 2, respectively, where population growth was moderate; Fritz et al., 2016). In southern Southeast Alaska and the central Gulf, natal dispersal of males was also greatest and adult female and male geographic ranges were largest (Jemison et al., 2018), suggesting similar causative factor(s).

Although our study demonstrates that Steller sea lions regularly delay weaning of their offspring past age 1 and that flexibility in this fitness parameter may result from optimal body growth patterns in different habitats, not directly related to population trend, more study is needed to understand life‐history strategies and trade‐offs in this species. To this end, studies underway to estimate age‐specific reproductive rates and to determine age‐specific costs of reproduction across the geographic range will be particularly informative. Studies of individual variation in reproductive performance and costs of reproduction are also needed to determine whether a significant proportion of adult females are currently nonreproductive in the west and whether maternal quality is shaping life‐history strategies in the endangered population.

## CONFLICT OF INTEREST

None declared.

## AUTHOR CONTRIBUTIONS

**Kelly K. Hastings:** Conceptualization (equal); data curation (equal); formal analysis (equal); funding acquisition (equal); investigation (equal); methodology (equal); project administration (equal); writing–original draft (lead); writing–review and editing (equal). **Devin S. Johnson:** Conceptualization (equal); formal analysis (equal); writing–review and editing (equal). **Grey W. Pendleton:** Conceptualization (equal); formal analysis (equal); methodology (equal); writing–review and editing (equal). **Brian S. Fadely:** Conceptualization (equal); investigation (equal); methodology (equal); writing–review and editing (equal). **Thomas S. Gelatt:** Conceptualization (equal); funding acquisition (equal); investigation (equal); methodology (equal); project administration (equal); writing–review and editing (equal).

## Data Availability

Data are available in the Dryad Digital Repository: https://doi.org/10.5061/dryad.3bk3j9kh5.
